# Antitumor Potential of Different Treatment Approaches Using Cold Atmospheric Pressure Plasma on Oral Squamous Cell Carcinoma Models: In Vitro Study

**DOI:** 10.3390/biomedicines13020443

**Published:** 2025-02-11

**Authors:** Ognjan Pavlović, Miloš Lazarević, Aleksandar Jakovljević, Nikola Škoro, Nevena Puač, Slavko Mojsilović, Maja Miletić

**Affiliations:** 1 School of Dental Medicine, University of Belgrade, dr Subotica 6, 11000 Belgrade, Serbia; milos.lazarevic@stomf.bg.ac.rs (M.L.); a.jakovljevic@stomf.bg.ac.rs (A.J.); 2 Institute of Physics, University of Belgrade, Pregrevica 118, 11080 Belgrade, Serbia; nskoro@ipb.ac.rs (N.Š.); nevena@ipb.ac.rs (N.P.); 3 Institute for Medical Research, University of Belgrade, Bulevar Oslobodjenja 18, 11000 Belgrade, Serbia; slavko@imi.bg.ac.rs

**Keywords:** cold atmospheric plasma, plasma-treated medium, antitumor therapy, oral squamous cell carcinoma, 3D cell models

## Abstract

**Background/Objectives:** Cold atmospheric plasma (CAP) has shown a strong anticancer effect on a variety of tumors, presenting a new approach for the effective treatment of oral squamous cell carcinoma (OSCC), one of the most prevalent malignant neoplasms with a high mortality rate. Here, we aimed to comprehensively investigate the antitumor potential of two approaches of CAP treatment on both two-dimensional and three-dimensional OSCC cell line models, as well as to analyze whether plasma treatment enhances the sensitivity of OSCC to chemotherapy. **Methods:** An in-house designed plasma needle, with helium as a working gas, was used to treat the SCC-25 cell line directly or indirectly via plasma-treated medium (PTM). The antitumor effect of CAP was assessed by measuring cell viability, apoptosis, adhesion, and migration. In addition, the combined effect of PTM and cisplatin was analyzed in SCC-25 tumor spheroids, as a more complex and reliable in vitro model. **Results:** Both plasma treatments showed time-dependent antitumor effects affecting their viability, adhesion, and migration. The rate of apoptosis was higher after incubation with PTM and is mediated by the intrinsic pathway. By utilizing the 3D spheroid carcinoma model, we confirmed the antitumor potential of CAP and additionally demonstrated an increased chemosensitivity of PTM-treated carcinoma cells. **Conclusions:** The results of our study illustrate a promising avenue for the application of CAP as a therapeutic option for OSCC, either as a standalone treatment or in combination with cisplatin.

## 1. Introduction

Head and neck cancer (HNC) is the seventh most common cancer globally, with squamous cell carcinoma (SCC) being the most frequent histological type [1,2]. While the 5-year survival rate for early-stage oral SCC (OSCC) ranges from 70% to 90%, it drops to about 50% for advanced-stage cases [3]. Although surgery represents the most effective treatment for head and neck cancer, the risk of recurrence remains high [4]. Other treatments for OSCC, such as radiation, cryosurgery, photodynamic therapy, and electro chemotherapy, have limited effectiveness and can cause serious side effects. Therefore, there is an urgent need for exploring new therapeutic approaches.

Recently, cold atmospheric plasma (CAP) has emerged as a promising tool for diverse applications in biomedicine, including cancer research and therapy [1,4,5,6,7,8,9]. CAP is a partially ionized gas composed of ions, electrons, reactive oxygen and nitrogen species (RONS), and UV photons. These components are also the main mediators of the CAP’s biological effects. Cold plasma operates at or near room temperature and is generated at atmospheric pressure, making it suitable for biomedical applications. In laboratory conditions, CAP can be generated using various techniques and different gasses, each with its own advantages and applications [10]. Although it is now well accepted that the anticancer effects of CAP are mainly due to the RONS it generates [11], there is still limited knowledge of the exact mechanisms of its effects on tumor cells, which has a major impact on the optimization and standardization of the CAP treatment protocols.

CAP exhibits remarkable versatility, as it can be directly applied in the treatment of superficial or luminal tumors and can also be utilized indirectly through plasma-treated water or other mediums [12,13,14,15,16]. Direct CAP application has some limitations due to the narrow possibility of delivering RONS to internal target tissues. In contrast, the indirect approach offers greater flexibility, as it activates RONS in various liquids, allowing for the treatment of targets regardless of their anatomic location [11]. Although different studies have shown the anticancer potential of CAP on different types of carcinomas under specific conditions [17,18,19,20,21,22], the data on the influence on oral malignancies [11,23,24,25,26] is limited, and the comparative effect of both types of plasma treatments still needs to be elucidated [27].

Furthermore, recent studies have demonstrated the potential of the synergistic therapeutic effect of CAP with existing clinical oncotherapies, such as radiation therapy, chemotherapy, targeted therapy, and immunotherapy, enhancing individualized treatment plans for patients [1,17,28,29]. This concept of combining therapeutic strategies holds clinical relevance in overcoming two major challenges in head and neck cancer (HNC) treatment: carcinoma cell resistance and severe side effects, which often result in incomplete therapy and disease recurrence and should be investigated thoroughly [30,31].

Although the most in vitro studies use a two-dimensional (2D) tumor model on the cell monolayer, three-dimensional (3D) culture methods, such as tumor spheroids, have increasingly been implemented in the preclinical testing of new therapeutics, as they mimic in vivo tumor behavior more closely [32]. These models replicate the physical and biochemical characteristics of a solid tumor mass, making the results derived from this tumor model more clinically relevant than those obtained from experiments using cell monolayers [33].

Therefore, this study aimed to evaluate and compare the antitumor potential of the direct and indirect application of CAP generated by a modified plasma needle as a plasma source, on both 2D and 3D in vitro tumor models using an OSCC cell line. In addition, we analyzed the combined effect of plasma-treated medium (PTM) and cisplatin, the standard adjuvant therapeutic used in OSCC treatment, in order to evaluate whether PTM could enhance the therapeutic efficacy and reduce the required doses of the chemotherapeutic.

## 2. Materials and Methods

### 2.1. CAP System for Treatments

The CAP source used for treatments was a modified plasma needle, developed and constructed in the Centre for Non-equilibrium Processes Institute of Physics, Belgrade. This device operates at 13.56 MHz using helium (He) as a working gas [34]. The plasma needle device used for cell medium treatments was investigated in detail by different diagnostic techniques and used for the treatment of human mesenchymal stem cells, bacteria suspensions, dentin surfaces, plant cells, etc. Previously, we performed detailed electrical characterizations of the plasma needle device with and without the samples, determination of the power deposited in plasma [34,35], emission imaging [36], mass spectrometry of reactive species (including ions) [37], temperature measurements, etc., and presented these in the literature [38,39]. As we could establish in different experiments, the plasma could be ignited by a free-standing source (without a grounded electrode), and the insertion of different biological targets only slightly influenced the plasma properties. This was also the case with direct cell treatments and the treatment of cell culture medium, where a change of target did not result in a modification in current and voltage waveforms or the power delivered to the discharge. A sine voltage signal produced by a Kurt J. Lesker-300 W power supply (providing 50 W of forwarded power) was brought to the powered electrode made of wolfram wire (with diameter 0.5 mm), which was enclosed in a ceramic tube so only the wire tip was exposed. Derivative probes were mounted onto the power line close to the plasma needle enabling real-time monitoring of the electrical waveforms and obtaining the power delivered to the plasma system. In all experiments, the power was around 2 W. The gas flow of He was kept constant at 1 slm (standard liter per minute). Tissue culture plates with 48 wells were used for all treatments. To facilitate the stability of CAP treatments, the wells were grounded by using copper tape placed at the outer side of the plate. Plasma plume was generated at the tip of the powered electrode forming a conic volume from the tip, with the cone base touching the treated surface. Visually observed, the formed discharge had the same shape and emission intensity for both types of targets. Previous optical emission measurements showed the presence of He lines, as well as N_2_ and O_2_ lines, in the discharge spectrum [40].

Two different protocols of CAP treatment were performed: direct and indirect. Direct plasma treatment involved the application of plasma directly to tumor cells previously seeded in wells. For indirect treatment, CAP was used to generate reactive species inside the cell culture medium, which was then placed in contact with the tumor cells. In this case, gas phase plasma chemistry and short-living RONS do not come into direct contact with cells. Instead, the reactive species produced in the plasma induce deposition of long-lived reactive species in the PTM, which then interact with tumor cells.

**Figure 1:**
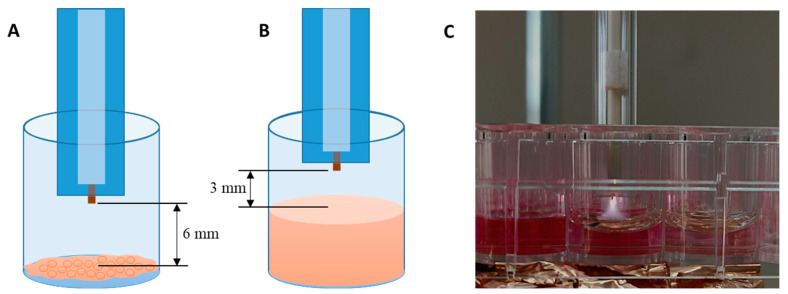
Positioning of the plasma needle in (**A**) direct treatments and (**B**) treatment of cell culture medium (PTM generation). (**C**) Photography of the plasma treating cell culture medium.

For the treatments, we used a distance of 6 mm from the cells for direct CAP treatment and a distance of 3 mm between the medium surface and the pin for indirect treatments (Figure 1). In both cases, the tube was positioned inside the well but, considering the diameter of the well, there was around 1.5 mm between the well walls and the glass tube to allow some inflow of the surrounding air, and mixing with He from the jet.

The plasma exposure times were 10 s, 30 s, and 60 s for direct treatments. Cell medium treatments (production of PTM) were performed for 30 s, 60 s, 120 s, and/or 180 s, depending on the experimental setup. Those exposure times were well defined through our previous scientific works [35]. Immediately after plasma treatment, concentrations of long-lived reactive species (H_2_O_2_, NO_2_^−^, NO_3_^−^) were assessed using a semi-quantitative measurement method based on colorimetric test strips. For H_2_O_2_, we employed MQuant 110011, for NO_2_^−^, we used MQuant 110057, and for NO_3_^−^, MQuant 1.10020. For pH measurement strips, MQuant 109,535 was used.

### 2.2. Cell Culture

The SCC-25 cell line (ATCC^®^ CRL-1628™) was used for the generation of both 2D monolayer cultures and 3D spheroid models. The cells were cultured in complete growth medium: Dulbecco’s modified Eagle’s medium (DMEM)/Ham’s F-12 (1:1) containing 1.2 g/L sodium bicarbonate, 2.5 mM L-glutamine, 15 mM HEPES, and 0.5 mM sodium pyruvate, supplemented with 400 ng/mL hydrocortisone, 100,000 IU/mL Penicillin, 100 mg/mL Streptomycin, and 10% fetal bovine serum (all from Thermo Fisher Scientific, Waltham, MA, USA). They were grown in a humidified atmosphere under standard conditions (37 °C and 5% CO_2_).

For 3D tumor models, ultra-low adherent flat-bottom 96-well microplates (Nunclon Sphera, Thermo Fisher Scientific) were used for obtaining the spheroids. In those plates, 10,000 cells were seeded per well, and the medium (3dGRO™ Spheroid Medium, Sigma-Aldrich, St. Louis, MO, USA) was changed every 3rd day until most of the obtained spheroids were of a desired size of minimum 300 nm. The size of spheroids was measured on CKX53 inverted phase-contrast microscope equipped with EP50 camera (Olympus, Tokyo, Japan).

### 2.3. Experimental Protocol

In the case of two-dimensional tumor models, 20,000 cells were seeded per well in a 48-well microtiter plate (Thermo Fisher Scientific). After 24 h of incubation, to allow cells to adhere, the cells in the monolayer were subjected to either direct or PTM treatment. Direct CAP treatment was performed well-by-well after temporary partial removal of medium (leaving 50 μL of medium over cells to avoid cell dehydration). Immediately after the treatment, the medium was returned, and further testing was conducted. For indirect treatment, DMEM/F-12 medium, without supplements (FBS, hydrocortisone and antibiotics), was treated with CAP and subsequently transferred into the corresponding wells. Thereafter, the supplements were added at the appropriate concentrations to make up the complete growth medium. After 24 h of incubation, specified assays were performed.

For the 3D tumor models, only the effect of PTM (3dGRO™ Spheroid Medium treated with CAP for 120 s and 180 s) was examined. PTM was transferred over the spheroids formed as previously described. Prior to the addition of PTM, most of the culture medium from each well was discarded, leaving only 50 μL per well to prevent the aspiration of present spheroids. Afterwards, either 180 μL, 120 μL, or 60 μL of PTM was added to the culture wells and filled with fresh medium up to a final volume of 240 μL to achieve the required concentration of the PTM of 75%, 50%, and 25%, respectively. To assess the combined effect of PTM and a chemotherapeutic drug, cisplatin (EBEWE Pharma Ges.m.b.H. Nfg.KG, Unterach, Austria) was used in increasing concentrations of 10 μM, 25 μM, 50 μM, 75 μM, and 100 μM.

### 2.4. Cell Viability (MTT)

To assess the viability of the SSC-25 cell line grown in a monolayer, we used 3-(4,5-dimethylthiazol-2-yl)-2,5-diphenyltetrazolium bromide (MTT) assay (Sigma-Aldrich). To examine the cytotoxicity of cold plasma following direct treatment, or 24 h after cultivation in PTM, the medium was replaced by standard growth medium, and MTT was added in a final concentration of 0.5 mg/mL. After 4 h of incubation in standard culture conditions, the medium was removed and the purple formazan crystals, formed by the activity of metabolically active cells, were dissolved in dimethyl sulfoxide. The optical density (OD) of the colored solution was measured at 540 nm using an automatic microplate reader (LabSystems Multiskan PLUS, Helsinki, Finland). Percentage of cell viability was calculated as the following ratio:

R = ((ODsample − ODblank)/(ODcontrol − ODblank)) × 100

### 2.5. Assessment of Apoptosis

To assess the programmed cell death in SCC-25 cells after both treatments, we performed an Annexin V assay, using a Dead Cell Apoptosis Kit with Annexin V Alexa Fluor™ 488 and Propidium Iodide (PI) (Thermo Fisher Scientific), as well as luminescence-based Caspase Glo assays (Promega, Madison, WI, USA). As a positive control for apoptosis, we used cisplatin in a 100 μM concentration.

Upon direct or PTM treatment of SCC-25, the cells were detached by 0.05% trypsin and 0.022% EDTA in phosphate-buffered saline (PBS) (Capricorn Scientific, Ebsdorfergrund, Germany). After detachment, the cells were washed in PBS and resuspended in annexin-binding buffer (provided in the kit) in a concentration of 1 × 10^5^ per 100 μL aliquot. The cells were than stained with Alexa Fluor™ 488 Annexin V (1:20) and PI (1 μg/mL) for 15 min at room temperature, and analyzed using a BD FACSCalibur flow cytometer (Becton Dickinson, Franklin Lakes, NJ, USA). Living cells were negative for both Annexin V and PI, early apoptotic cells stained positive for Annexin V only, and late apoptotic/necrotic cells stained positive for both Annexin V and PI.

The activity of caspases 3, 7, 8, and 9, as drivers of programmed cell death, was measured using the luminescence-based Caspase Glo^®^ 3/7, Caspase Glo^®^ 8, and Caspase Glo^®^ 9 assays (Promega) according to the manufacturer’s instructions. Briefly, SCC-25 cells were cultured in a white-walled 96-well plate in the control medium or in PTM. After 24 h of incubation in the standard culture conditions, caspase 3/7, caspase 8, or caspase 9 reagent was added to the respective wells of the culture plate in a volume equal to that of the culture medium (100 μL). After mixing and incubation at room temperature for 30 min, the luminescence was measured by a Wallac 1420 Victor 2 Microplate Reader (PerkinElmer, Waltham, MA, USA) in 15 min intervals for a total of 90 min, in order to detect the peak caspase activity.

### 2.6. Adhesion Cell Assay

The cells were seeded in a monolayer in a 48-well plate, as described previously, and allowed to adhere for 24 h under standard culture conditions. After the plasma treatment and 24 h incubation with PTM, non-adherent cells were removed by rinsing with PBS. Adherent cells were then fixed with 4% formaldehyde and stained with 0.2% crystal violet dye in PBS for 10 min. The wells were rinsed several times with PBS to remove excess dye and the residual cell-bound dye was dissolved with 33% acetic acid. The supernatants were transferred to a 96-well microtiter plate in triplicates, and the absorbance was measured at 540 nm using the automatic microplate reader. The results were then converted into the percentage of adherence using the same formula as for the percentage of viability, mentioned above.

### 2.7. Migration Assay

After reaching the confluence in a 2D culture, a scratch was made on a cell monolayer along the diameter of the well using a sterile pipette tip, and detached cells were rinsed with PBS. Following this, the cells were treated with CAP, or PTM was added to the wells. After the CAP treatment, fresh medium was added to the cells. At this time point (0 h), and after an additional 24 h incubation, a phase-contrast inverted microscope with a camera was used to observe and capture the migration rates. The chosen areas of interest were analyzed utilizing the Wound Healing Size Tool plugin for ImageJ software 1.48 (NIH, Bethesda, MD, USA). The technique enables the measurement of the lesion area (μm^2^) in images acquired during a wound healing assay. The calculation of the rate of cell migration was performed utilizing the equation that was previously documented [41].

### 2.8. Cytotoxicity Assay on 3D Spheroid Culture

To assess the viability of SSC-25 cell line grown in spheroid tumor models after PTM treatment alone or in combination with cisplatin, we used CellTiter-Glo^®^ Luminescent Cell Viability Assay (Promega, Madison, WI, USA), according to the manufacturer′s instructions. The method is based on the chemiluminescent detection and measurement of ATP in cells. After 24 h incubation in PTM or control medium, with or without increasing concentrations of cisplatin, spheroids were transferred to a white-walled 96-well plate (Thermo Fisher Scientific), and the CellTiter-Glo reagent was added in a volume equal to the volume of the culture medium (100 μL). After 15 min incubation at room temperature, the luminescence was measured using a Wallac 1420 Victor 2 Microplate Reader.

### 2.9. Statistical Analysis

All data from our experiments are expressed as mean ± SD. The means the different groups in the experiments were compared using ordinary one-way ANOVA with Tukey’s multiple comparisons test when the data were normally distributed, or Kruskal–Wallis test with Dunn’s multiple comparisons test for data that divert from the normal distribution. The normality of the data was assessed using the Kolmogorov–Smirnov test. All the test statistics were performed using GraphPad Prism software, version 8.0.1. A p-value less than 0.05 is considered statistically significant.

## 3. Results

### 3.1. Concentration of Reactive Oxygen and Nitrogen Species in Treated Medium

After the plasma treatment, we measured the pH of the treated mediums and the concentration of RONS. The plasma treatments did not influence the pH of the treated mediums (pH = 9). The measured RONS were similar in both mediums. In the case of medium used for 3D cell models, there was an initial NO_3_^−^ concentration of 10 mg/L in the untreated samples. The concentrations of reactive species after 120 s of plasma treatment were 10 mg/L (H_2_O_2_), 25 mg/L (NO_3_^−^) and 2 mg/L (NO_2_^−^), while after 180 s, the concentrations were 25 mg/L (H_2_O_2_), 25 mg/L (NO_3_^−^), and 2 mg/L (NO_2_^−^). The difference in RONS concentration with the increase in treatment time can be observed only in case of hydrogen peroxide, while NO_x_ concentrations stayed the same.

### 3.2. Direct and Indirect CAP Treatments Significantly Reduce SCC-25 Viability in an Exposure-Dependent Manner

**Figure 2:**
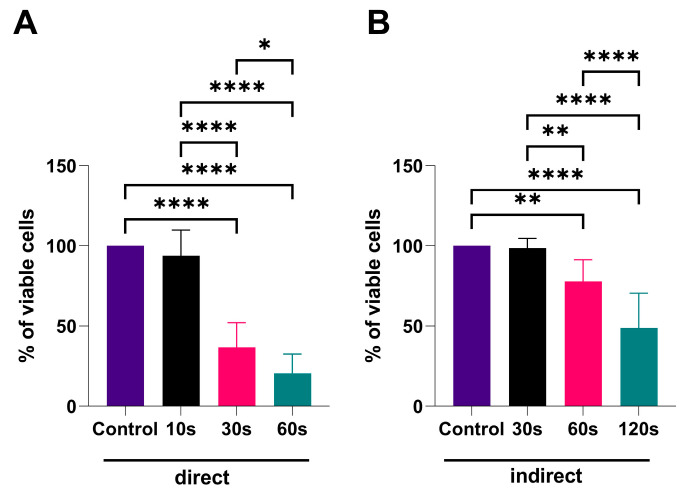
MTT assay results. The values are presented as the percentage of viable cells (normalized ratio of treated and control samples values). (**A**) Direct plasma treatment for 10 s, 30 s, and 60 s; (**B**) indirect plasma treatment (PTM exposed to CAP for 30 s, 60 s, and 120 s). The control group present the untreated cells grown in standard medium. The data are presented as mean ± SD, and statistical significance is denoted as * *p* < 0.05; ** *p* < 0.01; **** *p* < 0.0001.

The results from MTT assay, presented in Figure 2, revealed that both direct CAP treatment and applied PTM (indirect treatment) reduce the viability of the SCC-25, normalized to controls. The effect was progressively more pronounced, with an increase in CAP-treatment time. SCC-25 cells treated directly by CAP showed reduced viability, under 50% of the control, after 30 s (36.58%, *p* < 0.0001) and 60 s (20.54%, *p* < 0.0001) of plasma treatment. Additionally, statistically significant differences were noted between the treatment groups [10 s vs. 30 s (*p* < 0.0001), 10 s vs. 60 s (*p* < 0.0001) and 30 s vs. 60 s (*p* < 0.05)].

PTM also induced a linear decrease in the viability of cancer cells after 60 s (77.82%, *p* < 0.01) and 120 s (48.67%, *p* < 0.0001). However, a viability reduction of more than 50% was observed only with the longest treatment time (120 s). Similarly to direct CAP treatment, statistically significant differences were noted between the treatment groups [30 s vs. 60 s (*p* < 0.01), 30 s vs. 120 s (*p* < 0.0001) and 60 s vs. 120 s (*p* < 0.0001)].

### 3.3. Direct and Indirect Treatments Lead to Increased Apoptosis in SCC-25 Cells

**Figure 3:**
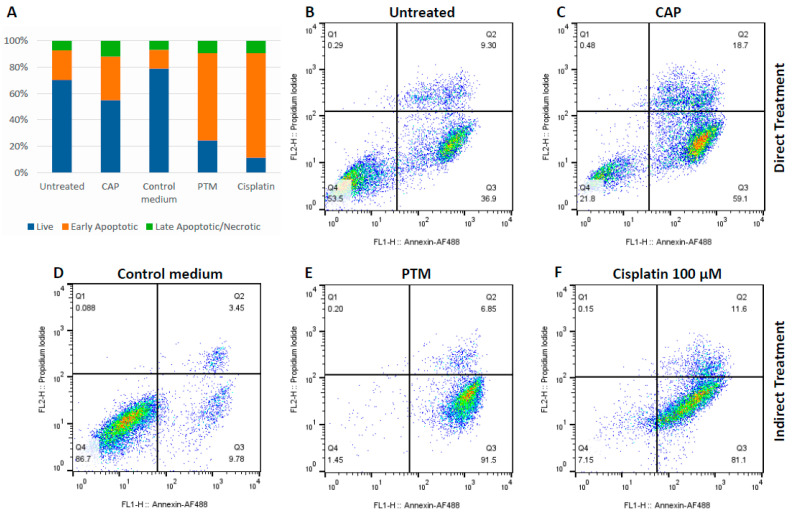
Flow cytometric screening of SCC-25 cells. PE Annexin-V staining was used to measure the apoptosis rate immediately after direct CAP treatment and 24 h after the incubation with PTM. (**A**) Stacked column diagram shows mean values of live vs. early apoptotic vs. late apoptotic/necrotic cell populations after different treatment protocols independently repeated three times. (**B**–**F**) The representative density plots for each treatment group are presented: (**B**) control untreated cells (direct treatment control); (**C**) cells treated directly with CAP; (**D**) cells incubated in control medium (indirect treatment control); (**E**) cells incubated with PTM; (**F**) cells treated with 100 μM of cisplatin (positive control).

Based on viability assay results, for the investigation of the mechanism of cell death induction, we have chosen the longest exposure to direct CAP (60 s), i.e., PTM obtained by 120 s CAP treatment. For the positive control, we treated cells with 100 μM cisplatin for 24 h. The results presented in Figure 3 show an increase in apoptotic cell population both after direct CAP and treatment with PTM.

The longest duration time of direct CAP treatment (60 s) led to an increase in apoptosis to 33% in comparison to the control untreated group (22%). After an incubation period of 24 h with PTM, the apoptosis rate was 66%, which was comparable to the results obtained with cisplatin as a positive control (75%). Both were much higher in comparison to the cells cultured in the control medium (14%) (Figure 3).

### 3.4. PTM Induces Caspase 9 Activation

To further investigate the process of apoptosis in cancer cells induced by plasma, we assessed the activity of caspases as drivers of programmed cell death. We measured the activity of two initiator caspases (8 and 9) and two effector caspases (3 and 7). Cells treated with 100 μM cisplatin served as a positive control. The relationship between the exposure of carcinoma cells to PTM and caspase activity was analyzed after 24 h incubation in PTM.

**Figure 4:**
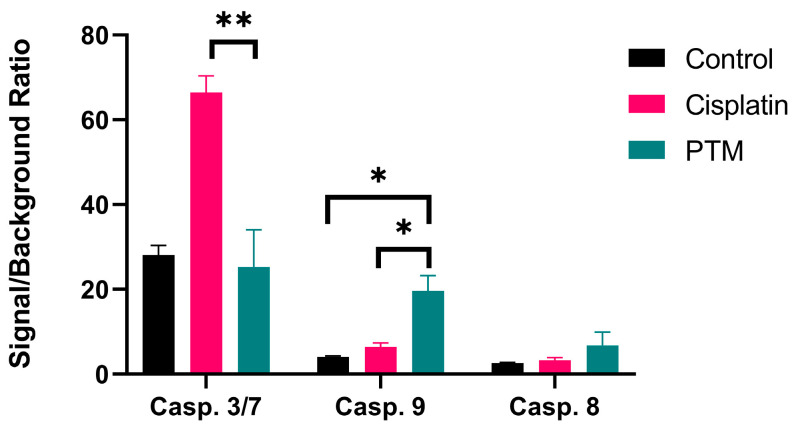
Caspase activation luminescence-based assay. The activation of different caspase systems (caspase 3/7, caspase 9, caspase 8) was measured 24 h after the incubation of SCC-25 cells with PTM obtained by 120 s exposure to CAP. Control cells were incubated in standard medium; positive control cells were treated with 100 μM of cisplatin. The data are presented as mean ± SD, and statistical significance is denoted as * *p* < 0.05; ** *p* < 0.01.

Figure 4 shows the caspase activities of cells incubated for 24 h with PTM, or with cisplatin (positive control), or in control medium (negative control). Caspase 3/7 activity increased over basal levels for cells treated with cisplatin, while treatment with PTM increased the activity of caspase 9. Neither treatment significantly increased the basal level of caspase 8 activity.

### 3.5. CAP Treatment Reduces Cell Adhesion in an Exposure-Dependent Manner

**Figure 5:**
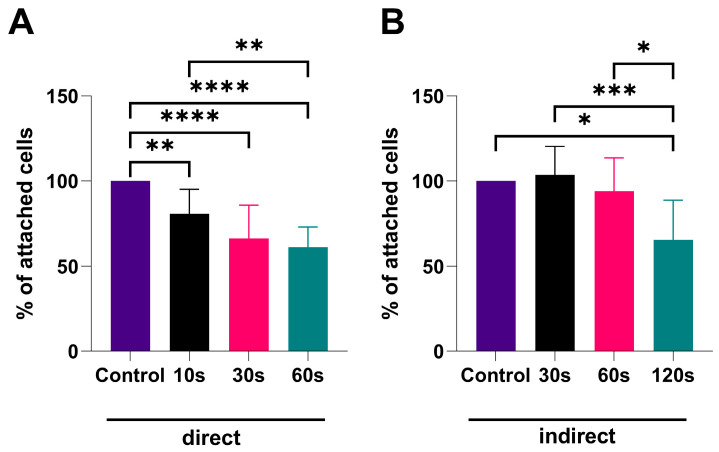
Adhesion assay results. The values are presented as percentages of adherent cells (normalized ratio of treated samples values with values of corresponding controls). (**A**) Direct CAP treatment; (**B**) indirect treatment with PTM; the data are presented as mean ± SD of three repeated experiments, and statistical significance is denoted as * *p* < 0.05; ** *p* < 0.01; *** *p* < 0.001; **** *p* < 0.0001.

The cell adhesion assay demonstrated a time-dependent decrease in the number of attached cells for both treatments (Figure 5). The statistically significant reduction in cell adhesion, compared to the control, was present after all exposure times for direct CAP treatment (Figure 5A). The percentage of attached cells was 80.67%, 66.33%, and 61.17% after 10 s, 30 s, and 60 s CAP treatment, respectively. Additionally, a significant difference between CAP treatment groups [10 s vs. 60 s (*p* < 0.01)] was noted.

The significant reduction in cell adhesion (65.45%) after the treatment with PTM was observed only after the longest exposure time (120 s) compared to the control (*p* < 0.05) (Figure 5B). Additionally, significant difference between PTM treatment groups (30 s vs. 120 s (*p* < 0.001), 60 s vs. 120 s (*p* < 0.05) was noted.

### 3.6. CAP Treatment Suppress Migration of SCC-25 in an Exposure-Dependent Manner

**Figure 6:**
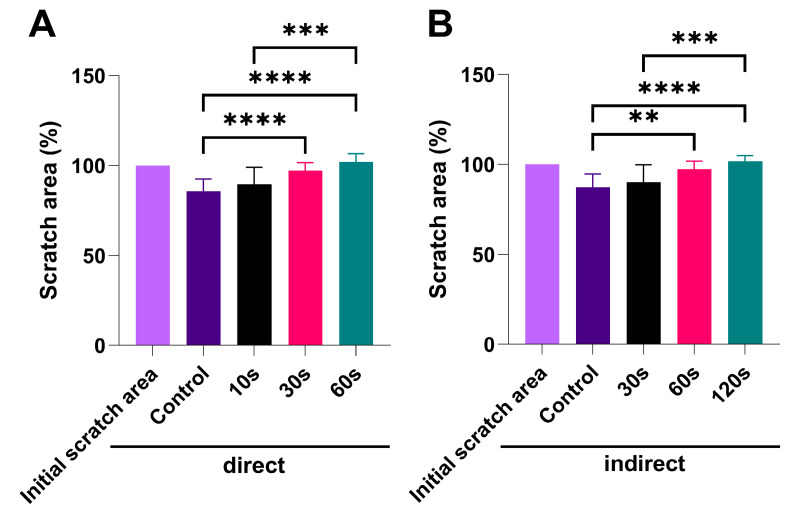
Wound healing assay results. (**A**) Direct CAP treatment; (**B**) indirect treatment with PTM; the data are presented as mean ± SD, and statistical significance is denoted as ** *p* < 0.01; *** *p* < 0.001; **** *p* < 0.0001.

To estimate the effect of CAP on cell migration in the SCC-25 cell line, wound healing assay analysis was performed, and the obtained results are presented in Figure 6. The results showed that CAP applied directly significantly suppressed the migration of SCC-25 cells across the denuded zone, in comparison to the untreated control cells, which migrated into the scratch area and reduced it to <90% of the initial void (Figure 6A). Similarly, 24 h incubation with PTM led to a linear decrease in cell migration capacity dependent on the exposure time used to produce PTM, with statistically significant effects for the longer (60 s and 120 s) exposure times compared to cells in control medium (Figure 6B).

### 3.7. PTM Reduces Viability and Increases Chemosensitivity of SCC-25 Cells in a 3D Spheroid Model

**Figure 7:**
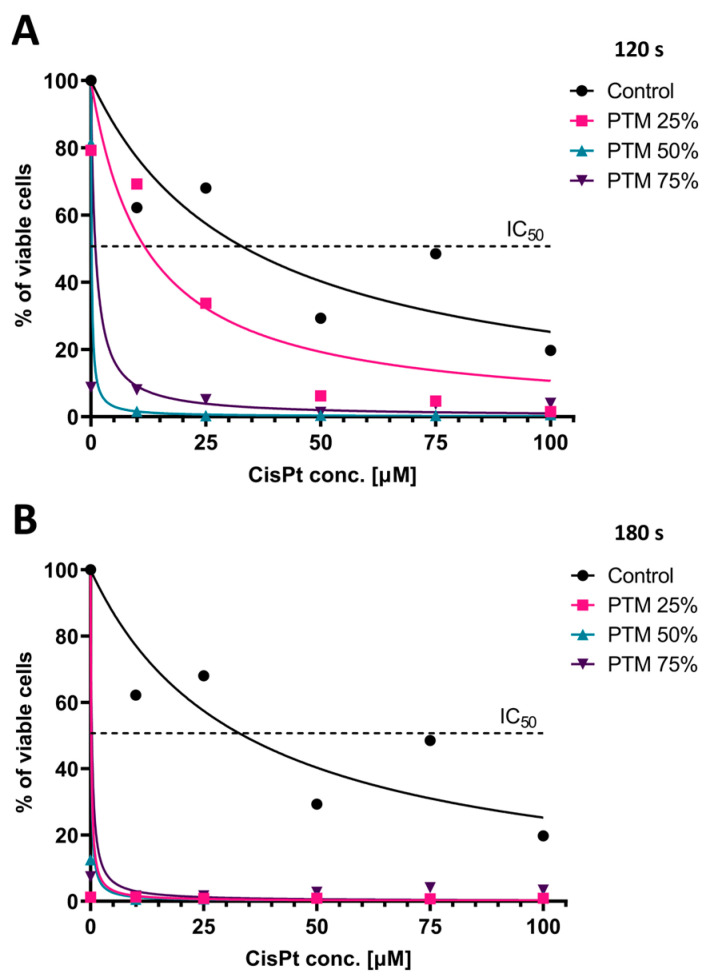
Cell titer Glo^®^ assay results in 3D spheroid models. The viability was measured after 24 h of incubation with PTM. (**A**) PTM treated for 120 s, alone or with increasing concentrations of cisplatin (0–100 μM); (**B**) PTM treated for 180 s, alone or with increasing concentrations of cisplatin (0–100 μM).

The results presented in Figure 7 illustrate the time and dose-dependent effects of PTM, as well as the dose-dependent and synergistic effects of cisplatin on SCC-25 spheroids. Spheroids were cultured for 24 h in the control medium or different proportions of PTM, namely, 25%, 50%, and 75%, where all samples had increasing concentrations of cisplatin (0–100 μM). The PTM was obtained by exposure to CAP for 120 s or 180 s. Cell viability, determined by a chemiluminescent assay based on the measurement of ATP in the cells, displayed a negative correlation with the increasing cisplatin concentration and the proportion of PTM in the culture medium. The half maximal inhibitory dose (IC50) of cisplatin in the control medium was 33.7 μM, while the combined treatment with 25% of PTM in the culture medium reduced IC50 to 11.9 μM, and even more (to 1 μM and less) with higher percentages of PTM. Moreover, PTM exposed to CAP for 180 s showed an even more pronounced effect on cell viability, as even the lowest proportion of this medium (25%) led to the death of most cells.

## 4. Discussion

We carried out a comprehensive in vitro investigation of the antitumor activity of CAP generated by an in-house designed plasma needle on an OSCC cell line. The results of our study show that both direct and indirect plasma treatments (treatment with PTM) have time-dependent effects on the viability, adhesion, and migration of tumor cells. While both treatments induced apoptotic cell death, the rate of apoptosis was higher after incubation with PTM, and is mediated by the intrinsic pathway. Importantly, this tumoricidal effect was also confirmed in a more complex and reliable 3D in vitro system. Moreover, plasma treatment increased the chemosensitivity of carcinoma cells in spheroids, highlighting the potential of CAP to minimize the therapeutic dose of cisplatin and associated side effects.

According to the results obtained by MTT assay, additionally supported by Annexin V staining, we showed that CAP treatment used in this study caused cytotoxic effects on OSCC after both direct and indirect treatments. The viability lower than 50% was associated with the longest exposure times in direct treatment and treatment with PTM. Our results indicate that direct CAP treatment is slightly more effective than treatment with PTM. Recently, Brunner et al. [23] showed that a half maximal inhibitory dose (IC_50_) was reached after 90 s or 120 s of direct exposure, depending on the tumor cell line tested. In our experiment, even after 30 s of direct CAP treatment, the viability of SCC-25 cells was below 50%. The reason for this may lay in the different types of cancer cells and the CAP source that was used. The plasma source used in their study was operating in air with high voltage pulses, thus resulting in the production of O_3_, which is not present in our case, exposing the sample to higher electric fields.

One of the main mechanisms by which cold plasma exerts its anticancer effect is an increase in the level of intracellular ROS in cancer cells. Compared to normal cells, it has to be noted that cancer cells are more sensitive to endogenous free radicals (ROS) and less efficient in repairing oxidative damage. This oxidative stress increases mitochondrial membrane permeability, triggering apoptosis, which is preferred over necrosis, as it prevents the inflammatory response associated with cell death. We analyzed the mechanism of the induced cell death for the longest CAP exposure times, and observed that PTM induces more apoptotic events in treated compared to untreated cells. Moreover, PTM induced apoptosis in two thirds of the cell population. In contrast, direct treatment with CAP led to more necrosis and left some viable cells, as well, which could be explained by the more localized direct effect supported by the existence of short-lived RONS. This pattern of direct plasma action on the cell monolayer, with clearly visible regions of cell debris and live cells, has already been described in our previous work [39]. On the other hand, the relatively high apoptosis rate in the control group for direct CAP treatment (Figure 3B) is possibly due to the suboptimal culture environment that the cells were subjected to while performing the direct plasma treatment (low volume of culture medium, room temperature, the environment outside the incubator). Consequently, we cannot exclude the impact of these culture conditions on the apoptosis rate in the direct CAP treatment group, as well. The uneven action of the plasma needle across the whole area of the well and the substandard culture conditions are probable reasons for the high variability in the apoptosis of directly treated cells.

A study conducted by Afrasiabi et al. [42] confirmed that plasma affects the mitochondrial pathway, leading to the activation of caspase-3, and consequently apoptosis, but they did not specifically measure the activation of all other caspases. To that end, we measured caspase activation levels following a 24 h incubation with PTM. The results showed that caspase-3 activation, the end phase of the caspase cascade, occurred only after incubation with cisplatin as a positive control. However, after treatment with PTM, there was a significant increase in caspase-9 activity in the carcinoma cells. Caspase-9 is a key player in the intrinsic or mitochondrial pathway [43]. On the other hand, caspase-8 is involved in receptor-mediated programmed cell death [44]. Therefore, it is not unexpected that we did not detect the activity of this caspase in our investigation, confirming there is no extrinsic pathway involved in these events. Based on these results, it can be suggested that PTM-induced RONS are involved in the activation of caspase-9, which in the next step activates caspase-3, followed by cell death. It could also be postulated that the PTM-induced caspase cascade starts later or takes some more time than the caspase cascade induced by cisplatin. Hence, at the time point when we measured the caspase activity (i.e., after 24 h of treatment), cisplatin-induced apoptosis reached the end stage, while PTM-induced apoptosis was at the initial phase of the intrinsic pathway.

Since the adhesive and migratory features of carcinoma cells play an important role in tumor behavior, we performed the adhesion and cell migration assay to explain how CAP affects it. Our results suggest that direct CAP treatment significantly affects the adhesion of SCC-25 cells, even after shorter exposure, while this effect was evident only with PTM treated for the longest period. By reducing the adhesiveness of the cells, we assume that the tumor cell′s carcinogenic potential is considerably interrupted. The other group of authors [45] observed the detachment of cells under the influence of CAP, but interestingly, they also monitored cell adhesion over time. Within the first two hours after the administration of PTM, they observed a strong decrease in cell adhesion. That could be explained by the fact that cancer cells undergo a change in morphology from a spread-out form to a contracted form within one hour after plasma treatment [45]. The exact mechanism of this action has not yet been elucidated. Another possible mechanism could be that CAP treatment leads to a reduction in adhesive molecules presented on the surface of the cells, which might be assessed in future investigations. The importance of disabling the migration of carcinoma cells is well known and of great importance to abrogate their invasive characteristics, which are closely associated with the local advancement of tumor or distant metastasis. Our results suggest that CAP inhibits the cell migration of the OSCC cell line, which is in accordance with the available scientific literature [46,47,48].

In contrast to cells grown in monolayers, as the most commonly used cell culture method, new in vitro 3D tumor models have been developed in recent years, allowing for the evaluation and examination of the tumor in a more in vivo-like setting [49]. This structure mimics the physical and biochemical features of a solid tumor mass, and thus, the results obtained on this type of tumor model should be of greater significance compared to the ones based on experiments conducted on cell monolayers.

To the best of our knowledge, there are currently no data in the literature regarding the antitumor effects of CAP on 3D OSCC. Furthermore, the issue of carcinoma cell resistance to chemotherapeutics prompted us to try combining PTM with cisplatin on a spheroid carcinoma model. Cisplatin is an alkylating drug that is one of the most used drugs in regular chemotherapy regimes to treat SCC [50]. Unfortunately, its use is connected to various side effects, including nephrotoxicity, ototoxicity, cardiotoxicity, neurotoxicity, and hepatotoxicity [50,51]. Our results have shown that PTM exposed to CAP for 120 s decreased the viability of tumor spheroids in a dose-dependent manner. Moreover, even the lowest percentage of this medium (25%) increased the susceptibility of cancer cells to cisplatin, while the higher percentages of PTM (50%, 75%) reduced the cell viability almost completely.

Recent studies of direct or indirect treatment with PTM in oncology have increasingly focused on combination therapies for reducing drug resistance and inducing synergistic cell death in different types of cancers [52,53,54,55]. The results of Young Joo Lee et al. [55], who examined the combinatory effect of PTM with the chemotherapeutic drug on two different ovarian cancer cell lines, showed a synergistic effect of PTM with cisplatin, which is in accordance with our results. They also revealed that there is a wide range of potential combinatory effects of chemotherapeutics with PTM, regarding the type of examining drug. For example, PTM combined with cisplatin induces synergistic toxic effects on ovarian spheroid models, which is in accordance with our results on OSCC spheroids, while the anti-proliferative effects were not increased when PTM was combined with doxorubicin and paclitaxel [55].

## 5. Conclusions

Our findings indicate that CAP generated by a modified plasma needle exerts an antineoplastic effect, either applied directly or via PTM. Both investigated treatment approaches have a time-dependent antitumor impact on OSCC cells, affecting their viability and their adhesive and migratory features. Nonetheless, PTM exhibits a more favorable tumoricidal effect by promoting apoptosis. Furthermore, PTM shows a potent synergistic effect in combination with cisplatin, ushering in a possible novel strategy for the management of SCC and other malignancies. Although further research is needed to corroborate these findings across multiple in vitro and in vivo models and to delve deeper into the underlying mechanisms of PTM/cisplatin-induced anticancer potential, our results illustrate a promising avenue for the application of PTM as a therapeutic option for cancer therapy, either as a standalone treatment or in combination with cisplatin.

## Data Availability

The additional data for this study are available from the corresponding authors upon reasonable request.
